# Supplementary material for: Gene therapy with feline anti-Müllerian hormone analogs disrupts folliculogenesis and induces pregnancy loss in female domestic cats
Source: Nat Commun. 2025 Feb 15;16:1668. doi: 10.1038/s41467-025-56924-5 (PMC11830062; doi:10.1038/s41467-025-56924-5)
Supplement: Supplementary file 1 — Supplementary Information [file 41467_2025_56924_MOESM1_ESM.pdf]

Supplementary Information

Gene therapy with feline anti-Müllerian hormone analogs disrupts folliculogenesis and induces pregnancy loss in female domestic cats

William A. Stocker[1]*, Lauren Olenick[2], Shreya Maskey[1,3], Denise Skrombolas[2], Haitong Luan[1], Sophie G. Harrison[1], Matt Wilson[2], Anne Traas[2], Mark Heffernan[2], Samantha Busfield[2], Kelly L. Walton[3] and Craig A. Harrison[1]*

[1]Department of Physiology, Monash Biomedicine Discovery Institute, Monash University, Clayton, VIC, Australia 3800; [2]Scout Bio, 601 Walnut St, Philadelphia, PA, USA 19106, [3]School of Biomedical Sciences, The University of Queensland, Brisbane, QLD, Australia 4072.

*Address correspondence to:

William A. Stocker, Monash Biomedicine Discovery Institute, Monash University, Clayton, VIC, Australia 3800, T: 61-3-9905-5132, E: william.stocker@monash.edu

Craig A. Harrison, Monash Biomedicine Discovery Institute, Monash University, Clayton, VIC, Australia 3800, T: 61-3-9905-5132, E: craig.harrison@monash.edu

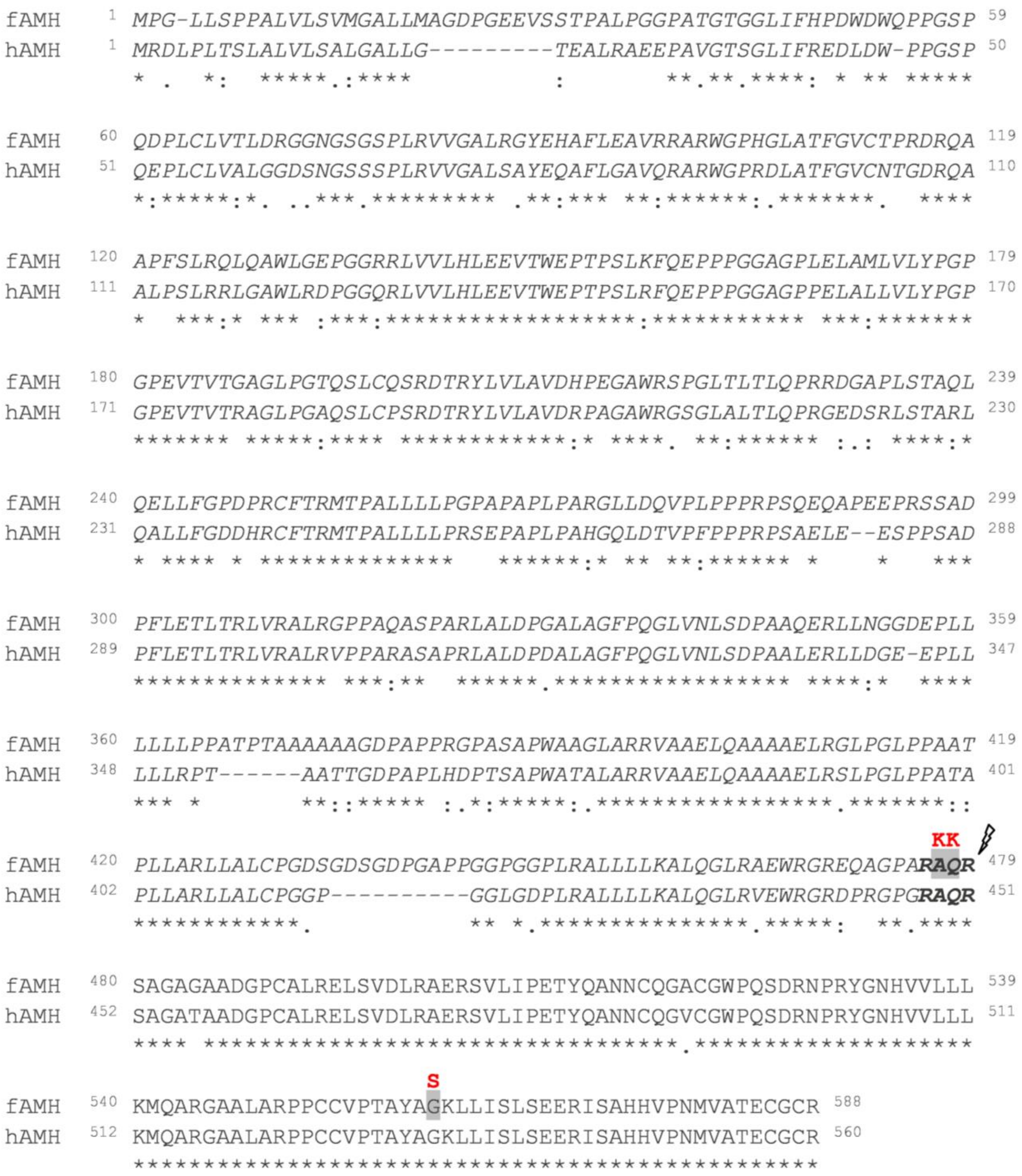

Supplementary Figure 1: Feline and human AMH sequence alignment. Sequences of feline (XP_011286375.2) and human (AAH49194.1) AMH were obtained from GenBank (https://www.ncbi.nlm.nih.gov/genbank/) and aligned using Clustal Omega (Conway Institute, University College Dublin, Dublin, Ireland). Prodomains are indicated by italics. The residues are numbered according to the first residue of the signal peptide. The cleavage recognition motif in both species 'RAQR', at the end of the prodomain, is in bold and the cleavage site marked (lightning bolt). Residues modified by *in vitro* site-directed mutagenesis in feline AMH are shaded, with the substitutions indicated above in red.

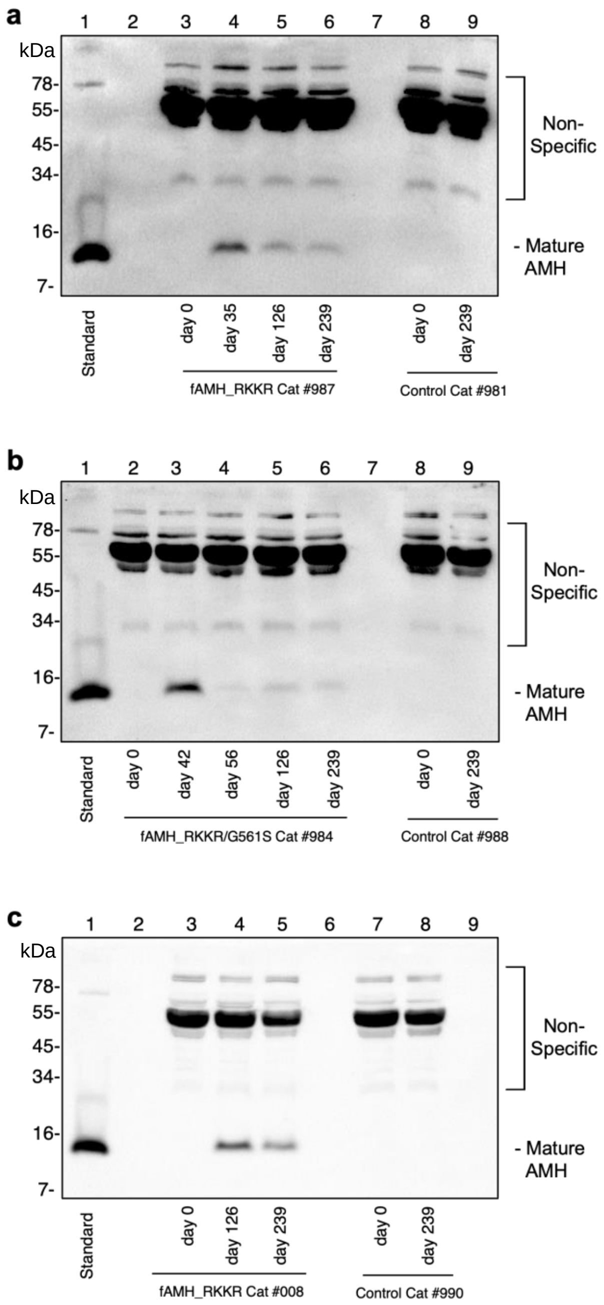

Supplementary Figure 2: *In vivo* processing of transgenic fAMH. Based on ELISA measurements, ~1 μl of serum collected at various times was diluted and analyzed by Western blotting, with samples run under reducing conditions. Blots were probed with mAb-5/6, targeted to the AMH mature domain. Recombinant mature human AMH was used as a positive control. (**a**) Serum collected on days 0, 35, 126 and 239 from cat #987 in the fAMH_RKKR group. Serum collected on days 0 and 239 from cat #981, in the control group, was run as a negative control. (**b**) Serum collected on days 0, 42, 56, 126 and 239 from cat #984 in the fAMH_RKKR/G561S group. Serum collected on days 0 and 239 from cat #988, in the control group, was run as a negative control. (**c**) Serum collected on days 0, 126 and 239 from cat #008 in the fAMH_RKKR group. Serum collected on days 0 and 239 from cat #990, in the control group, was run as a negative control. Similar results were obtained four times. Source data are provided as a Source Data file.

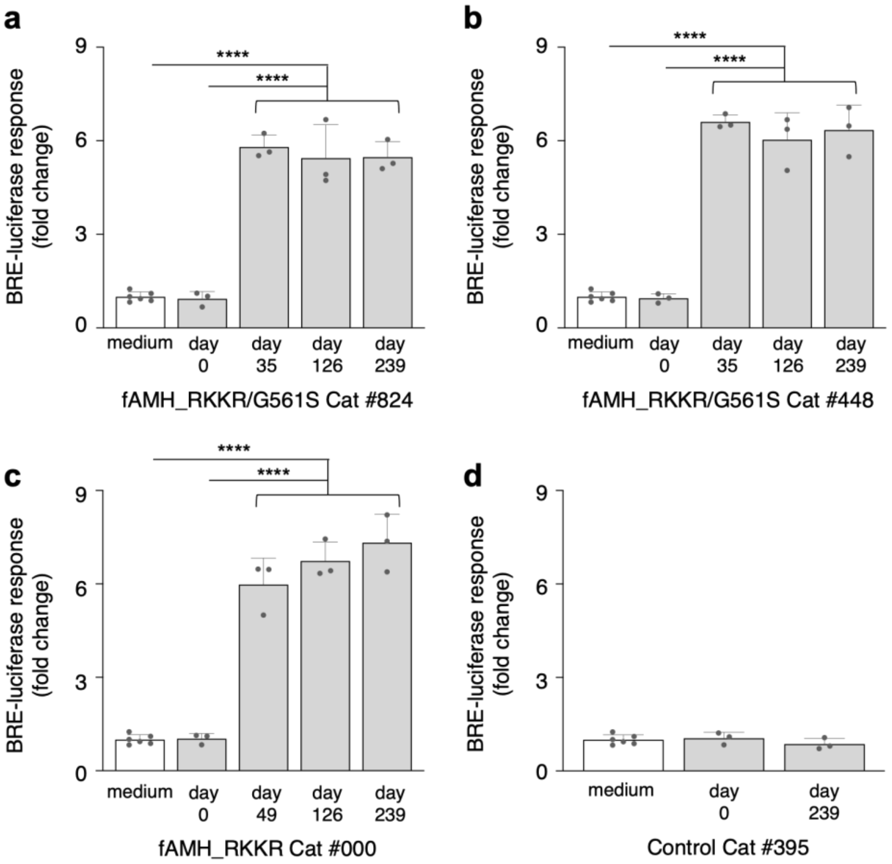

Supplementary Figure 3: Activity of transgenic fAMH. Serum from cats in the fAMH_RKKR/G561S (**a-b**) fAMH_RKKR (**c**) and control (**d**) groups was collected 2 weeks prior (day 0) and at different times (either 3- (day 35), 5- (day 49), 16- (day 126) or 32- (day 239) weeks) after AAV delivery. Based on AMH ELISA measurements, serum collected from four representative cats before or after AAV delivery was diluted 1:100 in fresh medium and used to treat HEK293T cells transfected with a SMAD1/5/9-responsive transcriptional reporter and AMH receptors. Luciferase activity for cells treated with diluted cat serum is presented as the mean ± SD of triplicates (*n*=3) from a representative experiment, relative to an adjusted value of 1.0 for the mean of the wells which received medium alone (*n*=6). The experiment was repeated 4 times. Data were analyzed using one-way ANOVA with Tukey's post hoc test (GraphPad Prism v.10). *P* < 0.0001 (****). Source data are provided as a Source Data file.

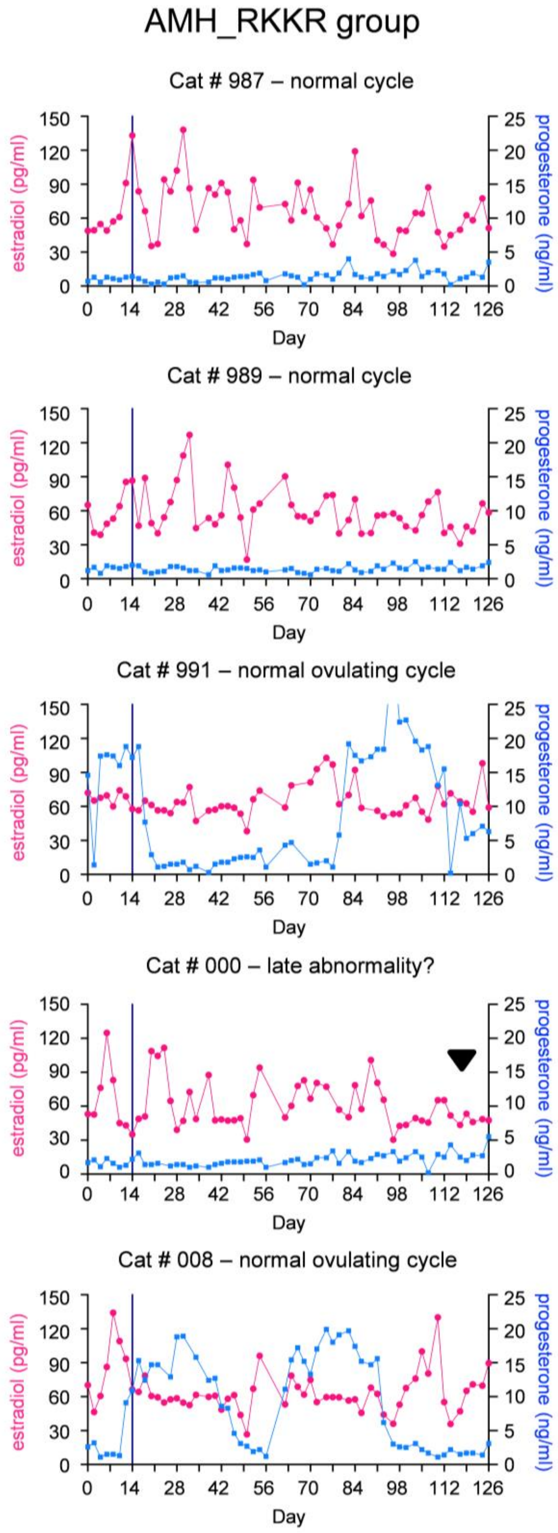

Supplementary Figure 4: Effect of elevated serum levels of fAMH_RKKR on estrus cycles and steroid hormone levels. Serum steroid hormone levels in cats (*n*=5) injected with AAV-fAMH_RKKR between days 0 and 126 of the study. Cyclic increases in estradiol levels (pink line) indicated estrus phases, while prolonged increases in progesterone levels (blue line) indicated luteal phases, following spontaneous ovulation. Potential abnormalities in estrus cyclicity in one cat in the AAV-fAMH_RKKR group is indicated (arrowhead). The day of AAV delivery (day 14) is indicated (vertical dark blue line). Source data are provided as a Source Data file.

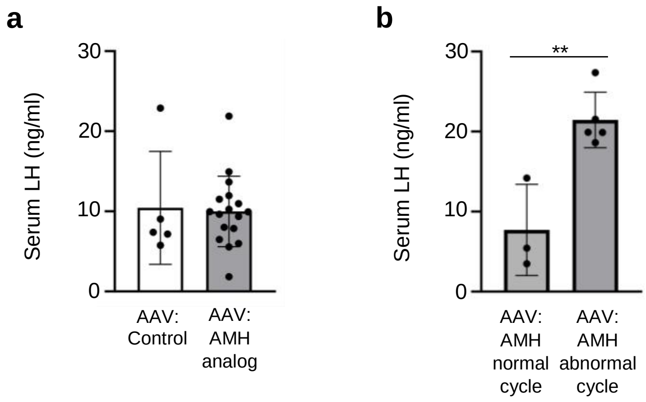

Supplementary Figure 5: Effect of elevated serum AMH on luteinizing hormone levels in cats. (**a**) Serum luteinizing hormone (LH) concentrations during estrus phase between study days 0 to 90, in samples collected from control (*n*=5 individual cats) and AMH overexpressing (*n*=17 samples, collected from 9 individual cats at different time points) cats. (**b**) Serum LH concentrations on day 126 in AMH overexpressing cats with normal (*n*=3 individual cats) and abnormal (*n*=5 individual cats) cycles. Bars represent mean ± SD of the mean. Data was analyzed using an unpaired two-tailed *t* test (GraphPad Prism v.10). *P* = 0.0049 (**). Source data are provided as a Source Data file.

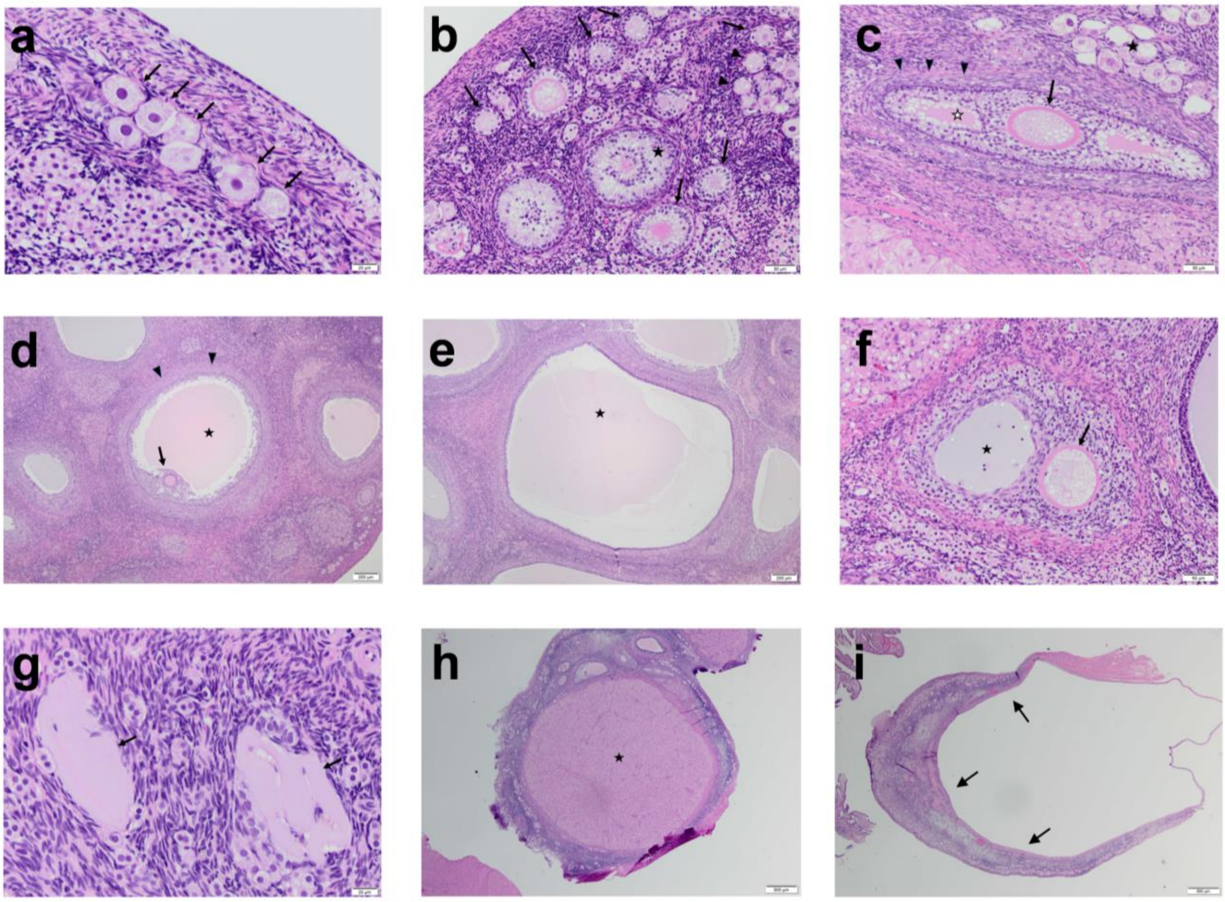

Supplementary Figure 6: Histological assessment of follicle stages in the feline ovary. Follicles were quantified by light microscopy. The following criteria were utilized to quantify follicular structures. (**a**) Primordial follicles: An oocyte surrounded by a single layer of flattened follicular cells (arrows). Cat #395. (**b**) Primary/secondary follicles: An oocyte surrounded by a single layer or multiple layers of cuboidal cells but with no evidence of space formation between the granulosa cells (arrows). (NB: Primordial follicles (arrowhead) and an early antral follicle (star) are also visible in this image). Cat #395. (**c**) Preantral follicle: An oocyte surrounded by multiple layers of granulosa cells and spaces have started to form in between the granulosa cells (white star) but a fully defined follicular antrum is not present. In this image, the oocyte/zona pellucida (arrow) and theca cell layer (arrowheads) are highlighted. (NB: A group of primordial follicles is also present (black star)). Cat #129. (**d**) Antral follicle < 2 mm in diameter: A fully formed follicular antrum is present. A corona radiata and cumulus oophorus is present (arrow). In this image, the follicular antrum (star) and theca cell layer (arrowhead) are highlighted. Cat #988. (**e**) Antral follicles > 2 mm in diameter: The follicular antrum (star) is indicated, although the oocyte is not present in this image. Cat #988. (**f**) Atretic follicle showing a degenerating oocyte with zona pellucida (arrow) and a shrinking/collapsing follicular antrum (star). Cat #395. (**g**) Remnant zona pellucida from an atretic follicle (arrows). Cat #988. (**h**) Mature corpus luteum (star). Cat #129. (**i**) A non-follicular cyst with inner aspect highlighted (arrows). Cat #985.

Supplementary Table 1: Weekly pregnancy observations between 3rd March 2022 (study day 211) and 12th May 2022 (study day 281).

Group	Cat #	Pregnancy Detected	Comments	Litter Born
CONTROL	981	Yes (7-Apr to 12-May 2022)	Detected via ultrasound (7-Apr-22)	Yes (20-May-22)
	986	Yes (21-Apr to 12-May 2022)		Yes (29-May-22)
	988	Yes (7-Apr to 12-May 2022)	Detected via ultrasound (7-Apr-22)<br>Bloody discharge (13-May-22)	No
	990	Yes (24-Mar to 5-May 2022)	Detected via ultrasound (24-Mar-22)<br>Enlarged nipples (31-Mar-22)	Yes (6-May-22)
	395	Yes (5-May to 12-May 2022)		Yes (12-Jun-22)
	134	Yes (24-Mar to 5-May 2022)	Detected via ultrasound, enlarged nipples (24-Mar-22)	Yes (6-May-22)
AMH_RKKR	987	No		No
	989	Yes (24-Mar to 28-Apr 2022)	Detected via ultrasound (24-Mar-22)<br>Ultrasound unclear but abdomen large (31-Mar-22)<br>Detected via ultrasound (7-Apr-22)<br>Ultrasound unclear (14-Apr-22)<br>Nothing detected via ultrasound (5-May-22)	No
	991	No		No
	000	Yes (28-Apr to 12-May 2022)	Detected (28-Apr-22)<br>Nothing detected (5-May-22)<br>Detected (12-May-22)	No
	008	Yes (28-Apr to 12-May 2022)		No
AMH_RKKR/G561S	983	Yes (14-Apr to 12-May 2022)	Detected via ultrasound (14-Apr-22)	No
	984	Yes (31-Mar to 5-May 2022)	Detected via ultrasound (31-Mar-22)<br>Ultrasound unclear (28-Apr-22)<br>Appears underdeveloped for length of pregnancy since first detection (5-May-22)<br>Nothing detected via ultrasound (12-May-22)	No
	985	Yes (21-Apr to 12-May 2022)	Detected (21-Apr-22)<br>Ultrasound unclear (5-May-22)<br>Detected (12-May-22)	No
	999	Yes (21-Apr to 12-May 2022)		No
	824	No		No
	448	Yes (5-May to 12-May 2022)		No
