# Supplementary material for: Gene therapy with feline anti-Müllerian hormone analogs disrupts folliculogenesis and induces pregnancy loss in female domestic cats
Source: Nat Commun. 2025 Feb 15;16:1668. doi: 10.1038/s41467-025-56924-5 (PMC11830062; doi:10.1038/s41467-025-56924-5)
Supplement: Supplementary file 3 — Reporting Summary [file 41467_2025_56924_MOESM3_ESM.pdf]

nature portfolio

Corresponding author(s): William A. Stocker and Craig A. Harrison

Last updated by author(s): 29th January 2025

Reporting Summary

Nature Portfolio wishes to improve the reproducibility of the work that we publish. This form provides structure for consistency and transparency in reporting. For further information on Nature Portfolio policies, see our Editorial Policies and the Editorial Policy Checklist.

Please do not complete any field with "not applicable" or n/a. Refer to the help text for what text to use if an item is not relevant to your study.
For final submission: please carefully check your responses for accuracy; you will not be able to make changes later.

Statistics

For all statistical analyses, confirm that the following items are present in the figure legend, table legend, main text, or Methods section.

n/a	Confirmed	
☐	☑	The exact sample size (n) for each experimental group/condition, given as a discrete number and unit of measurement
☐	☑	A statement on whether measurements were taken from distinct samples or whether the same sample was measured repeatedly
☐	☑	The statistical test(s) used AND whether they are one- or two-sided<br>*Only common tests should be described solely by name; describe more complex techniques in the Methods section.*
☑	☐	A description of all covariates tested
☑	☐	A description of any assumptions or corrections, such as tests of normality and adjustment for multiple comparisons
☐	☑	A full description of the statistical parameters including central tendency (e.g. means) or other basic estimates (e.g. regression coefficient) AND variation (e.g. standard deviation) or associated estimates of uncertainty (e.g. confidence intervals)
☐	☑	For null hypothesis testing, the test statistic (e.g. F, t, r) with confidence intervals, effect sizes, degrees of freedom and P value noted<br>*Give P values as exact values whenever suitable.*
☑	☐	For Bayesian analysis, information on the choice of priors and Markov chain Monte Carlo settings
☑	☐	For hierarchical and complex designs, identification of the appropriate level for tests and full reporting of outcomes
☑	☐	Estimates of effect sizes (e.g. Cohen's d, Pearson's r), indicating how they were calculated

Our web collection on statistics for biologists contains articles on many of the points above.

Software and code

Policy information about availability of computer code

Data collection	Data was collected using Bio-Rad Image Lab (version 4.1)
Data analysis	Data was analyzed using Microsoft Excel (2019), GraphPad Prism (v.10) and Clustal Omega (Webserver)

For manuscripts utilizing custom algorithms or software that are central to the research but not yet described in published literature, software must be made available to editors and reviewers. We strongly encourage code deposition in a community repository (e.g. GitHub). See the Nature Portfolio guidelines for submitting code & software for further information.

Data

Policy information about availability of data

All manuscripts must include a data availability statement. This statement should provide the following information, where applicable:

- Accession codes, unique identifiers, or web links for publicly available datasets
- A description of any restrictions on data availability
- For clinical datasets or third party data, please ensure that the statement adheres to our policy

A reporting summary and a source data file, containing the principle data presented in this article and its Supplementary Information, are available.
The accession codes and corresponding hyperlinks for the AMH sequences referred to in this study are
(feline: XP_011286375.2; https://www.ncbi.nlm.nih.gov/protein/XP_011286375.2) and
(human: AAH49194.1; https://www.ncbi.nlm.nih.gov/protein/AAH49194.1).
Source data are provided with this paper.

Research involving human participants, their data, or biological material

Policy information about studies with human participants or human data. See also policy information about sex, gender (identity/presentation), and sexual orientation and race, ethnicity and racism.

Reporting on sex and gender	This study did not involve human participants, their data, or biological material.
Reporting on race, ethnicity, or other socially relevant groupings	This study did not involve human participants, their data, or biological material.
Population characteristics	This study did not involve human participants, their data, or biological material.
Recruitment	This study did not involve human participants, their data, or biological material.
Ethics oversight	This study did not involve human participants, their data, or biological material.

Note that full information on the approval of the study protocol must also be provided in the manuscript.

Field-specific reporting

Please select the one below that is the best fit for your research. If you are not sure, read the appropriate sections before making your selection.

☑ Life sciences ☐ Behavioural & social sciences ☐ Ecological, evolutionary & environmental sciences

For a reference copy of the document with all sections, see nature.com/documents/nr-reporting-summary-flat.pdf

Life sciences study design

All studies must disclose on these points even when the disclosure is negative.

Sample size	We performed power calculations assuming a minimum mean difference between follicle counts of ≥ 50% (with standard deviations of ≥ 25%), with a probability of $P < 0.05$ (alpha level 5%), and power of 80% (beta level 20%). Using these values, we estimated that a minimum of n=4 cats would be sufficient to reach statistical significance in follicle counts between groups. For fertility measures, we predicted that the AMH vectored contraceptive would induce complete infertility, therefore larger mean differences were expected (with smaller deviations) and so n=4 cats was deemed powerful enough to capture all measures.
Data exclusions	One cat in the fAMH_RKKR group failed to express the transgene, therefore was excluded from the study. No other data was excluded from the study.
Replication	AAV injections were performed once per cat. One breeding trial lasting 12 weeks was performed. Transfections, Western blots to assess biosynthesis, and in vitro activity assays were performed on four separate occasions with conditioned medium or cat serum, respectively.
Randomization	Female cats were randomly assigned to treatment and breeding groups. The male cats were rotated amongst the groups at least every 2 weeks over a 12-week period. Sample allocation for in vitro studies was random.
Blinding	Investigators were not blinded to group allocations except for histologic evaluation, where the specimens were blinded in terms of treatment and control groups and assessed by a board-certified veterinary pathologist/theriogenologist.

Behavioural & social sciences study design

All studies must disclose on these points even when the disclosure is negative.

Study description	Behavioural and social sciences were not part of this study.
Research sample	Behavioural and social sciences were not part of this study.
Sampling strategy	Behavioural and social sciences were not part of this study.
Data collection	Behavioural and social sciences were not part of this study.
Timing	Behavioural and social sciences were not part of this study.
Data exclusions	Behavioural and social sciences were not part of this study.
Non-participation	Behavioural and social sciences were not part of this study.
Randomization	Behavioural and social sciences were not part of this study.

Ecological, evolutionary & environmental sciences study design

All studies must disclose on these points even when the disclosure is negative.

Study description	Ecological, evolutionary and environmental sciences were not part of this study.
Research sample	Ecological, evolutionary and environmental sciences were not part of this study.
Sampling strategy	Ecological, evolutionary and environmental sciences were not part of this study.
Data collection	Ecological, evolutionary and environmental sciences were not part of this study.
Timing and spatial scale	Ecological, evolutionary and environmental sciences were not part of this study.
Data exclusions	Ecological, evolutionary and environmental sciences were not part of this study.
Reproducibility	Ecological, evolutionary and environmental sciences were not part of this study.
Randomization	Ecological, evolutionary and environmental sciences were not part of this study.
Blinding	Ecological, evolutionary and environmental sciences were not part of this study.

Did the study involve field work? ☐ Yes ☑ No

Field work, collection and transport

Field conditions	This study did not involve field work.
Location	This study did not involve field work.
Access & import/export	This study did not involve field work.
Disturbance	This study did not involve field work.

Reporting for specific materials, systems and methods

We require information from authors about some types of materials, experimental systems and methods used in many studies. Here, indicate whether each material, system or method listed is relevant to your study. If you are not sure if a list item applies to your research, read the appropriate section before selecting a response.

Materials & experimental systems

n/a	Involved in the study	
☐	☑	Antibodies
☐	☑	Eukaryotic cell lines
☑	☐	Palaeontology and archaeology
☐	☑	Animals and other organisms
☑	☐	Clinical data
☑	☐	Dual use research of concern
☑	☐	Plants

Methods

n/a	Involved in the study	
☑	☐	ChIP-seq
☑	☐	Flow cytometry
☑	☐	MRI-based neuroimaging

Antibodies

Antibodies used	Primary: anti-AMH mAb-5/6 (Abcam, catalog # ab24542), used at 1:5000<br>Secondary: Amersham ECL sheep-derived horseradish peroxidase-conjugated anti-mouse IgG (Cytiva, catalog # NA931-1ML), used at 1:10000
Validation	Anti-AMH mAb-5/6 was validated when first produced (DOI: 10.1093/molehr/gah015,).

Eukaryotic cell lines

Policy information about cell lines and Sex and Gender in Research

Cell line source(s)	HEK293T cell line from Merck (catalog # 12022001-1VL)
Authentication	STR profiling was performed by Merck prior to purchase.
Mycoplasma contamination	Negative
Commonly misidentified lines (See ICLAC register)	No commonly misidentified cell lines were used in this study.

Palaeontology and Archaeology

Specimen provenance	
Specimen deposition	
Dating methods	

☐ Tick this box to confirm that the raw and calibrated dates are available in the paper or in Supplementary Information.

Ethics oversight	

Note that full information on the approval of the study protocol must also be provided in the manuscript.

Animals and other research organisms

Policy information about studies involving animals; ARRIVE guidelines recommended for reporting animal research, and Sex and Gender in Research

Laboratory animals	Domestic cats (Felis catus) were sourced from LFM Quality Labs, Inc. (USA). Female cats were between 19.5 months and 51 months old at the start of study. Male cats were 13 months old when introduced into the study.
Wild animals	This study did not involve wild animals.
Reporting on sex	The findings of this study are only applicable to females. Transgenic overexpression of AMH analogs was not performed on males.
Field-collected samples	No field-collected samples were used in this study.
Ethics oversight	The study protocol (21SCB021) was assessed by LFM Quality Laboratories, Inc. (USA) internal Institutional Animal Care and Use Committee (IACUC), and complies with all relevant ethical regulations.

Note that full information on the approval of the study protocol must also be provided in the manuscript.

Clinical data

Policy information about clinical studies

All manuscripts should comply with the ICMJE guidelines for publication of clinical research and a completed CONSORT checklist must be included with all submissions.

Clinical trial registration	
Study protocol	
Data collection	
Outcomes	

Dual use research of concern

Policy information about dual use research of concern

Hazards

Could the accidental, deliberate or reckless misuse of agents or technologies generated in the work, or the application of information presented in the manuscript, pose a threat to:

No	Yes	
☐	☐	Public health
☐	☐	National security
☐	☐	Crops and/or livestock
☐	☐	Ecosystems
☐	☐	Any other significant area

Experiments of concern

Does the work involve any of these experiments of concern:

No	Yes	
☐	☐	Demonstrate how to render a vaccine ineffective
☐	☐	Confer resistance to therapeutically useful antibiotics or antiviral agents
☐	☐	Enhance the virulence of a pathogen or render a nonpathogen virulent
☐	☐	Increase transmissibility of a pathogen
☐	☐	Alter the host range of a pathogen
☐	☐	Enable evasion of diagnostic/detection modalities
☐	☐	Enable the weaponization of a biological agent or toxin
☐	☐	Any other potentially harmful combination of experiments and agents

Plants

Seed stocks

Novel plant genotypes

Authentication

ChIP-seq

Data deposition

☐ Confirm that both raw and final processed data have been deposited in a public database such as GEO.

☐ Confirm that you have deposited or provided access to graph files (e.g. BED files) for the called peaks.

Data access links
May remain private before publication.

Files in database submission

Genome browser session
(e.g. UCSC)

Methodology

Replicates

Sequencing depth

Antibodies

Peak calling parameters

Data quality

Software

Flow Cytometry

Plots

Confirm that:

- [ ] The axis labels state the marker and fluorochrome used (e.g. CD4-FITC).
- [ ] The axis scales are clearly visible. Include numbers along axes only for bottom left plot of group (a 'group' is an analysis of identical markers).
- [ ] All plots are contour plots with outliers or pseudocolor plots.
- [ ] A numerical value for number of cells or percentage (with statistics) is provided.

Methodology

Sample preparation	
Instrument	
Software	
Cell population abundance	
Gating strategy	

- [ ] Tick this box to confirm that a figure exemplifying the gating strategy is provided in the Supplementary Information.

Magnetic resonance imaging

Experimental design

Design type	
Design specifications	
Behavioral performance measures	

Imaging type(s)	
Field strength	
Sequence & imaging parameters	
Area of acquisition	

Diffusion MRI ☐ Used ☐ Not used

Preprocessing

Preprocessing software	
Normalization	
Normalization template	
Noise and artifact removal	
Volume censoring	

Statistical modeling & inference

Model type and settings	
Effect(s) tested	

Specify type of analysis: ☐ Whole brain ☐ ROI-based ☐ Both

Statistic type for inference

(See Eklund et al. 2016)

Correction

Models & analysis

n/a	Involved in the study
☐	☐ Functional and/or effective connectivity
☐	☐ Graph analysis
☐	☐ Multivariate modeling or predictive analysis

Functional and/or effective connectivity

Graph analysis

Multivariate modeling and predictive analysis

This checklist template is licensed under a Creative Commons Attribution 4.0 International License, which permits use, sharing, adaptation, distribution and reproduction in any medium or format, as long as you give appropriate credit to the original author(s) and the source, provide a link to the Creative Commons license, and indicate if changes were made. The images or other third party material in this article are included in the article's Creative Commons license, unless indicated otherwise in a credit line to the material. If material is not included in the article's Creative Commons license and your intended use is not permitted by statutory regulation or exceeds the permitted use, you will need to obtain permission directly from the copyright holder. To view a copy of this license, visit http://creativecommons.org/licenses/by/4.0/
